# Application of the Nova food classification to the 2017-2018 Household Budget Survey: monitoring adherence to the recommendations of the *Dietary Guidelines for the Brazilian Population*

**DOI:** 10.1590/S2237-96222025v34e20240369.en

**Published:** 2025-05-12

**Authors:** Gabriela Lopes da Cruz, Giovanna Calixto Andrade, Fernanda Rauber, Renata Bertazzi Levy, Maria Laura da Costa Louzada

**Affiliations:** 1 Universidade de São Paulo, Faculdade de Saúde Pública, Departamento de Nutrição, São Paulo, SP, Brazil; 2 Universidade de São Paulo, Núcleo de Pesquisas Epidemiológicas em Nutrição e Saúde, São Paulo, SP, Brazil; 3 Universidade de São Paulo, Faculdade de Medicina, Departamento de Medicina Preventiva, SP, Brazil

**Keywords:** Food and Nutritional Surveillance, Nutrition Surveys, Dietary Guidelines, Nova Classification, Cross-Sectional Studies, Vigilancia Alimentaria y Nutricional, Encuestas Nutricionales, Guías Alimentarias, Clasificación Nova, Estudios Transversales

## Abstract

**Objective:**

To describe the food categorization method of the 2017-2018 Household Budget Survey as per the Nova classification, bringing transparency and replicability to the process of monitoring adherence to the recommendations of the *Dietary Guidelines for the Brazilian Population*.

**Methods:**

The foods reported in the Survey were classified in four stages, namely: identification of culinary preparations and items composed of more than one food; determination of the recipe for culinary preparations and items to be disaggregated; application of the Nova classification; sensitivity analysis.

**Results:**

After disaggregation, 1,856 items were classified according to the Nova classification, consisting of 1,050 unprocessed or minimally processed foods, 54 processed culinary ingredients, 160 processed foods and 592 ultra-processed foods. Foods whose classification raised questions during the accounted for 4% of the total dietary energy. The contribution of ultra-processed food to total caloric intake varied from 19.7% (95% confidence interval [95%CI] 19.3; 20.1) to 17.7% (95%CI 17.4; 18.1) after conducting sensitivity analysis.

**Conclusion:**

Using a standardized method to apply the Nova classification to the Household Budget Survey was effective and led to estimates whose uncertainties minimally affected the overall results. The methodology presented can be replicated in future editions of the Household Budget Survey and other food consumption studies, strengthening food and nutritional surveillance as applied to the Dietary Guidelines.

## Resumen

**Objetivo:**

Describir el método de categorización de alimentos de la Encuesta de Presupuesto Familiar 2017-2018 según la clasificación Nova, aportando transparencia y replicabilidad al proceso de seguimiento de la adhesión a las recomendaciones de la *Guía Alimentaria para la Población Brasileña*.

**Métodos:**

Se trató de clasificar los alimentos reportados en la Encuesta en cuatro fases. Fueron: identificación de preparaciones culinarias y elementos compuestos por más de un alimento, determinación de la receta de preparaciones culinarias y elementos a desagregar, aplicación de la clasificación Nova y análisis de sensibilidad.

**Resultados:**

Después de la desagregación, se clasificaron 1.856 artículos según la clasificación Nova, los cuales 1.050 correspondían a alimentos frescos o mínimamente procesados, 54 a ingredientes culinarios procesados, 160 a alimentos procesados ​​y 592 a alimentos ultraprocesados. Los alimentos cuya clasificación planteaba dudas representaban el 4% de la energía de la dieta. La participación calórica de los alimentos ultraprocesados ​​varió del 19,7% (intervalo de confianza del 95% [IC95%] 19,3; 20,1) al 17,7% (IC95%: 17,4; 18,1) después del análisis de sensibilidad.

**Conclusión:**

El uso de un método estandarizado para aplicar la clasificación Nova a la Encuesta de Presupuesto Familiar fue efectivo y condujo a estimaciones cuyas dudas impactaron mínimamente en el resultado. La metodología presentada podrá replicarse en futuras ediciones de la Encuesta y otros estudios de consumo de alimentos, fortaleciendo la vigilancia alimentaria y nutricional aplicada a la Guía Alimentaria.

## Introduction

The 2017-2018 Household Budget Survey (*Pesquisa de Orçamentos Familiares*) (1) presented, for the first time, a report on the personal food consumption of the Brazilian population, based on the Nova food classification (2). This classification divides foods into four groups according to the extent and purpose of the processing to which they have undergone. The groups are: unprocessed or minimally processed foods, processed culinary ingredients, processed foods and ultra-processed foods.

Assessment of food consumption as per the Nova classification enables monitoring of the population’s adherence to the recommendations of the *Dietary Guidelines for the Brazilian Population* (3), published in 2014. The Guidelines recommends: “Always prefer natural or minimally processed foods and culinary preparations to ultra-processed foods” (3). Adherence to the Guidelines’s recommendations benefits the population’s health, contributes to the prevention of chronic diseases and promotes environmental sustainability. The Guidelines is the official instrument of the Ministry of Health that supports public food and nutrition policies and directs actions within the country’s health services, which makes assessment of adherence essential.

Food surveys that collect data using 24-hour dietary recalls that are non-specific to capturing detailed information about the food’s degree of industrial processing present challenges in classifying some items according to the Nova classification. In these cases, it is necessary to disaggregate culinary preparations (meals made from minimally processed foods and culinary ingredients, made at home or in restaurants) into their underlying ingredients, so that these individual ingredients, rather than the entire preparation, are classified. Some food items, depending on their composition, can be assigned to different groups of the Nova classification (such as bread and cheese, which can be processed or ultra-processed), which also constitutes a challenge.

Considering the growing relevance of the Nova classification and the various possibilities for utilizing data from the Household Budget Survey, it is expected that several studies will be carried out using them as analysis tools. The Nova classification is expected to be applied in future editions of the Survey, as well as in other dietary surveys. The objective of this article was to describe the food categorization method of the 2017-2018 Household Budget Survey as per the Nova classification. We sought to bring transparency and replicability to the process of monitoring adherence to the recommendations of the *Dietary Guidelines for the Brazilian Population*.

## Methods

### Design

This is the description and assessment of the methodology used to classify the 2017-2018 Household Budget Survey, as per the Nova classification. 

### Background and study size

We used data from the personal food consumption module of the Household Budget Survey, conducted by the Brazilian Institute of Geography and Statistics between July 2017 and July 2018. The Survey used a complex two-stage sampling process. The first stage involved census tract clustering with geographic and socioeconomic stratification followed by random selection. In the second stage, households in the selected census tracts were randomly selected. Further details about the Survey can be found in a previous publication (1).

The Household Budget Survey databases are available at: https://www.ibge.gov.br/estatisticas/sociais/saude/24786-pesquisa-de-orcamentos-familiares2.html?edicao=28523&t=downloads.

### Participants and variables

Information on individual food consumption was collected from a subsample of 20,112 households, with information provided by residents aged 10 or over. In total, 46,164 individuals were selected to answer 24-hour dietary recall on two non-consecutive days. Participants were asked about all foods and drinks consumed the day before the interview. After identifying the items consumed, the interviewers requested details about the type of preparation of each food (when applicable) and the quantity consumed. 1,591 food items were recorded. Considering the different forms of preparation associated with these items (1 – roasted, 2 – cooked with fat, 3 – cooked without fat, 4 – raw, 5 – breaded, 6 – stewed, 7 – fried, 8 – grilled/barbecued, 9 – braised and 99 – undetermined), the number of food items reported reached 2,534. 

### Measurement

Food identification according to the Nova classification was carried out in four stages, applied by two nutritionists researchers specializing in ​​food consumption and familiar with the Household Budget Surveys. At all stages, uncertainties regarding food classification were discussed collaborativelywith other researchers knowledgeable about the Nova classification and Brazilian eating habits until consensus was reached. 

### Stage 1 – Identification of culinary preparations and items composed of more than one food

The first stage consisted of identifying items that needed to be disaggregated in order to be classified, so as to be able to identify the ingredients that comprised them (4). Disaggregation is necessary to classify individual ingredients of culinary preparations (such as *feijoada* [a dish consisting of bean and meat stew], *baião de dois* [a dish consisting of rice, beans and meat] and cakes, as well as braised, fried and breaded preparations) and composite food items (combined items, such as couscous with milk or açaí with guaraná and granola) according to the Nova classification. 

Culinary preparations typical of Brazilian food culture (such as rice, beans, pasta, moqueca, etc.) and foods prepared using methods involving the addition of ingredients (cooked with fat, breaded, stewed, fried and braised) were identified for disaggregation. Foods with a single ingredient that was raw, roasted or cooked without fat were not selected for disaggregation. 

The following are examples of items selected for disaggregation:

- Item name: Rice (polished, parboiled, long-grain, short long-grain)
- Type: culinary preparation
- Disaggregated into the following items: rice, garlic, onion, soybean oil and salt
- Item name: Passion fruit juice
- Type: culinary preparation
- Disaggregated into the following items: passion fruit, water and sugar
- Item name: Coffee with milk
- Type: composite food item
- Disaggregated into the following items: coffee and milk

Items that require disaggregation were differentiated from industrially manufactured products (food composed of several industrially pre-combined and processed ingredients), which can be processed or ultra-processed foods. These items were identified through information contained in their names, such as the food brand (Tang and Ovomaltine) and through the terms “diet” and “light”, “ready to eat” and “industrialized”. Even though they comprise multiple ingredients, such items are not disaggregated, as per the Nova classification definition, and are therefore allocated to stage 3 to be classified (2). As an example, ready-to-eat farofa (coarse seasoned and fried cassava flour) and light ready-to-eat farofa were identified as industrialized items and selected for stage 3. Farofa with eggs, banana farofa and kale farofa were categorized as culinary preparations and selected for disaggregation. 

In the case of items with non-specific descriptions, we consulted external sources to determine the most likely alternative. The main approach in these cases was to compare, based on these sources, the frequency of availability of foods that could be both culinary preparations and industrially manufactured products, identifying those with the highest occurrence. Examples of external sources included:

- Consulting food purchase data from the Household Budget Survey module on household food availability in Brazil (5) conducted in the same period, comparing availability and frequency of purchases of the same foods with different forms of preparation. For example: in the case of lasagna, the frequency of purchasing “lasagna pasta” – indicative of homemade lasagna preparation – was compared with purchasing “frozen lasagna” – indicative of industrialized food.
- Consulting retail chain food sales websites, checking the availability of the same foods with different degrees of processing. For example: in the case of fruit juices, the presence/absence in retail outlets of items such as tamarind juice, cupuaçu juice, beetroot juice and kale juice, either bottled or in aseptic packaging, was checked. Absence/low frequency indicated their being categorized as culinary preparations made from fruit.
- checking the Nova classification groups to which the ingredients of a food item eligible for disaggregation would belong if it were disaggregated, eliminating the need for disaggregation if they all belonged to the same group. For example: the item hamburger, consisting of hamburger buns, beef burgers, ketchup and mayonnaise – all ultra-processed foods – did not require disaggregation.

### Stage 2 – Determination of the recipe for culinary preparations and items to be disaggregated

Culinary preparations and composite food items selected in stage 1 were disaggregated into the ingredients that compose them and corresponding quantities, using standardized recipes from the University of São Paulo Brazilian Food Composition Table (*Tabela Brasileira de Composição de Alimentos da Universidade de São Paulo*), version 7.0 (available at: http://www.fcf.usp.br/tbca). 

### Stage 3 – Application of the Nova classification

All foods (including items that were not disaggregated and ingredients comprising those that were disaggregated) were categorized according to the Nova classification into the following groups: unprocessed or minimally processed foods, processed culinary ingredients, processed foods and ultra-processed foods (2). To this end, definitions and examples of these groups were used (Table 1) as per Monteiro et al. (2).

**Table 1:** Definition and examples of Nova classification food groups, according to the degree and purpose of industrial processing

Nova classification group	Definition	Examples
**Unprocessed or minimally processed foods**	Unprocessedfoods: fresh foods; edible parts of plants, animals, fungi and algae, which have undergone little or no change in relation to their state in nature. Minimally processed: unprocessed foods that have undergone minimal modifications, which include removing inedible parts, drying, crushing, grinding, roasting, cooking with water, pasteurization, refrigeration, freezing, packaging or vacuum packaging. Vitamins and minerals can be added to foods in this group, usually to replace nutrients lost during processing.	Cereals, legumes, vegetables, fruits, seeds, leaves, stems, roots and tubers, fresh meat (muscle meat and offal), eggs, milk, flour. They also include foods composed of two or more items from this group, such as mixed dried fruit, granola made from cereals, nuts and dried fruit without added sugar, honey or oil, pasta, couscous and polenta made with flour and water, and fortified foods such as wheat or corn flour with added iron and folic acid.
**Processed culinary ingredients**	Extraction of foods from the first group or directly from nature through processes such as pressing, extraction, centrifugation and refining. These processes create products used to season and prepare foods from the first group in dishes prepared from scratch. Additives are generally not necessary and are rarely found in this group.	Sugar (granulated, caster, brown and demerara), molasses, honey, vegetable oils, animal fats (such as butter and lard), corn starch, vinegar and salt. Sugar (granulated, caster, brown and demerara), molasses, honey, vegetable oils, animal fats (such as butter and lard), corn starch, vinegar and salt. They also include products made up of two items from the group, such as salted butter, and items with added vitamins or minerals, such as iodized salt.
**Processed foods**	They include foods produced by adding a processed culinary ingredient (such as salt, sugar, oils or fats) to unprocessed or minimally processed foods. They are relatively simple processing products, using conservation methods such as canning and bottling, non-alcoholic fermentation and cooking or baking to form bread and cheese. These processes and ingredients aim to increase durability and complexify the sensorial qualities of foods. Processed foods often contain additives that extend the life of the product, preserve its properties or inhibit the proliferation of microorganisms (such as preservatives and antioxidants). They do not contain additives with a cosmetic function.	Bread, cheese, pickled legumes and vegetables, salted or sugar sweetened nuts and seeds, fruit in syrup and jellies. Dried and cured meats, dried or canned fish. Fermented alcoholic beverages like beer and wine. When made exclusively with foods from the first two groups, pasties, cakes, cookies, ready-to-heat products such as burgers and pies, pasta and pre-prepared dishes.
**Ultra-processed food**	Industrial formulations resulting from different stages and types of processing. The following are markers of ultra-processed foods: a) cosmetic food additives that modify the sensorial characteristics of the product, such as flavorings, colorings, artificial sweeteners, emulsifiers, thickeners, flavor enhancers and emulsifying agents; and/or b) substances for industrial use, rarely used in cooking, such as fructose, high-fructose corn syrup, invert sugar, maltodextrin, dextrose, lactose, added gluten, hydrogenated or interesterified oils, hydrolyzed proteins, protein isolate and mechanically separated meat.	Soft drinks, fruit drinks, energy drinks, milk drinks and flavored yogurts. Mass-produced packaged bread, sweet and savory biscuits. Candies, chocolates and sweets in general, margarine, nuggets, sausages, hamburgers and other reconstituted meat products, breakfast cereals, instant noodles, powdered soups,sauces. Ready-to-eat products such as pre-prepared burgers, pies, pasta and pizza.

Food items corresponding to a single Nova classification group, such as fresh fruits and vegetables, eggs, fresh meat, vegetable oil, soft drinks, margarine, cookies and artificial juices, were assigned to the equivalent group. For other foods that could contain variations, information contained in the description field was used for decision making. 

For example, foods with names indicating that they were fresh were allocated to the unprocessed or minimally processed group (fresh tuna, fresh pork, fresh mushroom, peanuts in natura). Canned foods were classified as processed foods (canned sweetcorn, canned hearts of palm and canned sardines). Items indicated as being “diet” and “light”, “ready-to-eat” and “industrialized” were classified as ultra-processed. 

Natural yogurt and low-fat yogurt were allocated to the unprocessed or minimally processed group. Yogurt of any flavor, light yogurt of any flavor and diet yogurt of any flavor were classified as ultra-processed foods. White bread and homemade bread were identified as processed foods. Industrialized bread of any brand, including diet bread and light bread were classified as ultra-processed foods. When the item’s name specified the food brand, we searched the ingredient list to classify it accordingly. 

Another strategy was to use information from food sales websites to guide the classification of items that, depending on their composition, could belong to different groups of the Nova classification (such as cheese, coconut milk and bacon, which can be processed or ultra-processed). The ingredient lists of different brands of the same food available in retail outlets were examined and the food was then categorized according to the most frequent Nova classification group among them. Using this approach, items such as cream and granola were classified as ultra-processed due to the presence of substances for industrial use and cosmetic additives (Table 1) in most products on sale on the Brazilian market. 

Characteristics of Brazilian culture and eating habits were also considered when making decisions, whereby classification was made according to the most common alternative. This was the case of fruit preserves, such as marmalade, guava paste and banana paste, all of which were classified as processed foods.

### Stage 4 – Sensitivity analysis

After consulting external sources and group discussion, foods still considered to be of uncertain classification in stages 1 and 3 were submitted to a group decision regarding classification. These foods were selected for sensitivity analyses, evaluating how variation in their classification could affect the results. Differences in estimates were assessed based on overlapping confidence intervals.

### Statistical analysis

Data from the first day of dietary recall was used, as the information collected was of higher quality and aligned with the analytical standard adopted in the report (1). The quantities of each food and drink reported in the Survey were converted into grams or milliliters and calories using the Brazilian Food Composition Table. The proportion of calories relative to total dietary energy was calculated. Sensitivity analysis reclassified foods with uncertain classification to the alternative group, by evaluationg the overlap of the confidence intervals of the means. 

## Results

**Figure 1:**
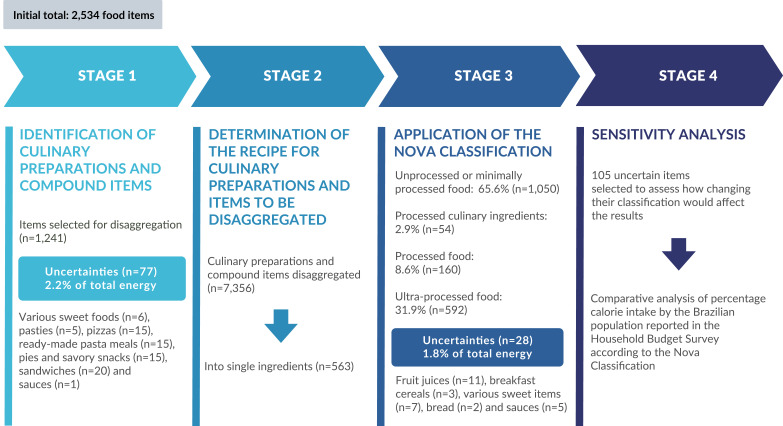
Method used to categorize foods reported in the Household Budget Survey according to the Nova classification. Brazil, 2017-2018 (n=46,164)

The four stages outlined in the methodology, together with their results, are illustrated in the flowchart below (Figure 1).

In stage 1, 1,241 items (49%) were selected for disaggregation, while 1,293 items (51%) remained non-disaggregated and were directly allocated to stage 3. Among those selected for disaggregation, 77 were considered uncertain due to the impossibility of identifying them clearly either as culinary preparations or as industrially manufactured products. These items, together, accounted for 2.2% (95%CI 1.9; 2.6) of the total calories consumed by the Brazilian population and included: various sweet foods (n=6), pasties (n=5), pizzas (n=15), ready-made pasta meals (lasagna, cannelloni) (n=15), pies and savory snacks (coxinha, pie, croquette, kibbeh) (n=15), sandwiches (n=20) and sauces (n=1) (Table 2). 

**Table 2:** Food items reported in the Household Budget Survey identified as uncertain in stages 1 and 3 of the methodology. Brazil, 2017-2018 (n=46,164))

Items identified as “Uncertain”	
**Stage 1** – **identification of culinary preparations and items composed of more than one food** (n=77)	**Stage 3** – **Application of the Nova classification** (n=28)
*Americano* (ham, cheese, egg, bacon, lettuce and tomato toasted sandwich) Fried cheese cake Fried cassava cake Fried cod fish cake Brigadeiro dessert Chicken cannelloni Cheese cannelloni Cheese and ham cannelloni Ricotta cannelloni Meat capeletti Chicken capeletti Coxinha Croquette Peanut butter Puff pastry slice Small individual pies (cheese, meat, shrimp, etc.) Large pies (cheese, chicken, shrimp, hearts of palm, etc.) Sfiha Meat sfiha Chicken sfiha Cheese sfiha Ricotta sfiha Four-cheese lasagna Maria-Mole (marshmallow) Meringue Mille-feuille *Minipastel* (mini fried pasty) Tomato sauce Fried pasty with sweet filling Baked wholemeal pasty (cheese, meat, hearts of palm, etc.) Baked pasty (cheese, meat, hearts of palm, etc.) Custard pie Pizza Pepperoni pizza Chicken pizza with catupiry Smoked sirloin pizza Buffalo mozzarella and sun-dried tomato pizza Fish pizza (tuna, salmon, sardines) Sweet pizza of any flavor Margherita pizza (mozzarella, tomato and basil) Mozzarella pizza Neapolitan pizza Portuguese pizza Ham pizza Vegetarian pizza Pepperoni pizza Cheese pizza Kibbeh Rissoles (cheese, meat, shrimp, etc.) Rondelli Shrimp rondelli Meat rondelli Cheese and ham rondelli Bauru sandwich Tuna sandwich Roast beef sandwich Pork sandwich Fillet sandwich Chicken sandwich Mortadella sandwich Turkey breast sandwich Ham sandwich Cheese sandwich (unspecified) Minas cheese sandwich Yellow cheese sandwich Yellow cheese and ham sandwich Roast beef sandwich Salami sandwich Sardine sandwich Natural sandwich Ham, cheese and tomato sandwich Vegetarian sandwich Savory pies of any flavor Shrimp yakisoba Beef yakisoba Chicken yakisoba Vegetable yakisoba	**Individual non-disaggregated items**: Buttered peanuts Rice cream Milk cream Fruit jelly of any brand or flavor Granola Coconut milk Marshmallow Cereal mix Tomato sauce Peanut candy Corn bread Cheese bread Jawbreaker Juice Guava juice Orange juice Mango juice Peach juice Peach juice syrup Grape juice Peanut nougat **Following disaggregation**: Cashew, juice, concentrated, bottled Milk cream Orange, pear, juice, unsweetened Orange, juice, unsweetened, Citrus sinensis Milk, condensed, with sugar, canned Sauce, pepper, tabasco Grape, juice, concentrated, bottled

After group discussion, the items were mostly kept as non-disaggregated, based on the premise that their main components, or those with the highest caloric contribution, would belong to the same Nova classification group, justifying their being analyzed jointly.

In stage 2, culinary preparations and composite food items were disaggregated using standardized recipes, resulting in 7,356 ingredients, 563 of which were unique unrepeated ingredients. This was expected since the same ingredients appeared in different recipes.

In stage 3, after disaggregation, 1,856 items were organized according to the Nova classification into its four groups, with 1,050 (56.6%) items identified as unprocessed or minimally processed foods, 54 (2.9%) as processed culinary ingredients, 160 (8.6%) as processed foods and 592 (31.9%) as ultra-processed foods. 

In the case of 28 items, which accounted for 1.8% (95%CI 1.7; 1.9) of the total caloric intake reported in the Survey, information contained in food names was insufficient to identify characteristics of industrial processing, so that they were allocated as uncertain items. These items included: fruit juices (n=11), breakfast cereals (granola and cereal mix) (n=3), sweet items (n=7), bread (n=2) and sauces (n=5) (Table 2). 

Uncertainty with regard to these items derived from lack of specification in the description of the Survey items regarding the degree of processing. This was especially relevant for correctly identifying items such as fresh or processed cream and natural or processed juices. Further uncertainty arose from the fact that some foods, such as ready-made jellies and sauces, may belong to different groups of the Nova classification depending on the list of their ingredients, which varies between brands and can determine their classification as processed or ultra-processed.

Foods with descriptions that included terms such as “unspecified” also generated uncertainty, when there was uncertainty about the food’s condition (whether it was fresh, frozen or canned), and whether it was sweetened or salted. Items that generated uncertainties were mostly classified as ultra-processed. Foods described as “unspecified” were classified based on the most frequently consumed similar item. For example, “unspecified” bread was classified as the French white bread, the most consumed type of bread in the Survey, falling into the processed food group. 

In stage 4, items identified as uncertain in stages 1 and 3 were selected for sensitivity analysis. Using the methodology described above, according to the Nova classification, unprocessed or minimally processed foods accounted for 53.4% ​​(95%CI 53.0; 53.8), processed culinary ingredients for 15.6% (95%CI 15.4; 15.8), processed foods for 11.3% (95%CI 11.1; 11.5) and ultra-processed foods for 19.7% (95%CI 19.3; 20.1) of the total caloric intake of the Brazilian population. 

**Figure 2:**
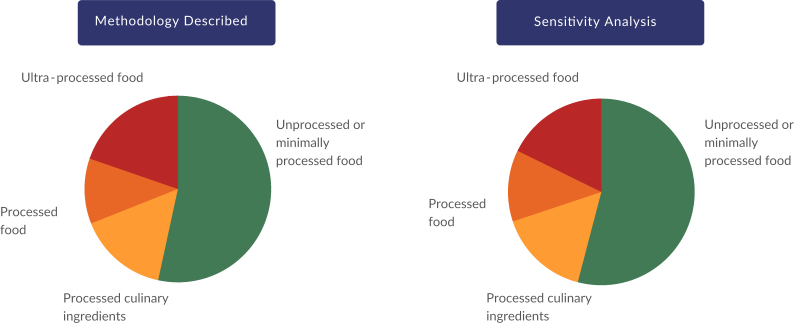
Results of the sensitivity analysis considering the share of Nova classification food groups in relation to total calories consumed by the Brazilian population aged 10 years and over. Brazil, 2017-2018 (n=46,164)

The sensitivity analysis consisted of reclassifying all foods that caused uncertainty to the non-selected classification group. After this analysis, unprocessed or minimally processed foods accounted for 54.1% (95%CI 53.7; 54.4), processed culinary ingredients for 15.8% (95%CI 15.6; 16.0), processed foods for 12.4% (95%CI 12.2; 12.7) and ultra-processed foods for 17.7% (95%CI 17.4; 18.1) of the population’s total calorie intake (Figure 2).

The main changes in the sensitivity analysis included switching foods from the ultra-processed food group to the processed food group. There was also an increase in the amount of natural fruit juices and a decrease in the amount of artificial juices. All sandwiches were disaggregated, reducing the category of ultra-processed snacks. A decrease in the quantity of ultra-processed sweets was observed, which were selected for disaggregation in the sensitivity analysis.

## Discussion

The use of a standardized method was effective in categorizing the Household Budget Survey according to the Nova classification, even though the Survey undertook data collection with non-specific dietary recall to capture the degree of food processing. This methodology, applied by a team knowledgeable about Brazilian eating habits, resulted in few uncertainties, minimally impacting the results obtained.

Food and nutritional surveillance is a fundamental part of the National Food and Nutrition Policy. The aim is to generate data for continuous monitoring and evaluation of the population’s dietary and nutritional profile, essential for informing public policies and guiding actions in health services. The Household Budget Survey stands out as a pivotal database due to its national representativeness, ensured by the participation of more than 40,000 individuals from urban and rural areas from different regions of the country. 

Following the publication of the *Dietary Guidelines for the Brazilian Population* in 2014, the Survey became a fundamental instrument for monitoring the population’s adherence to its recommendations by means of analyses using the Nova classification. It is expected that the methodology presented in this study will be replicated in future editions of the Survey, thus continuing the monitoring process. 

The Household Budget Surveys involve different sets of food data, such as individual food consumption, household food availability and food security, and well-characterized socioeconomic information. Combining these data enables systemic approaches to studying nutrition, which is something that is increasingly urgent in order to address problematic global health problems such as obesity, malnutrition and climate change (6). 

Analyses and discussions based on the Nova classification are increasingly relevant. Consistent evidence has shown an association between the consumption of ultra-processed foods and the risk of more than 30 adverse health outcomes, including obesity and other chronic non-communicable diseases, as well as mortality from all causes (7). This problem is particularly important in Brazil due to the global trend of increasing ultra-processed food consumption in populous and middle-income countries, particularly in Latin America (8).

The method used in this study made it possible to evaluate in detail the consumption of ultra-processed foods in different population groups. This approach can be replicated to gain a better understanding of consumption patterns and their public health implications. Standardization of this method in different contexts can help identify socioeconomic variables that influence food consumption, thus contributing to the development of more effective health promotion strategies.

The methodology described for identifying foods according to the Nova classification in the Household Budget Surveys was applied in studies to generate data to monitor the quality of the Brazilian population’s diet. Data on household food purchasing revealed an increasing trend in the availability of ultra-processed foods in Brazil over the last three decades, concomitantly with the decline in unprocessed and minimally processed foods (9). Food consumption data showed that ultra-processed foods account for a fifth of the Brazilian diet, with significant differences in consumption according to socioeconomic status (10). The use of this methodology also enabled identification of association between consumption of ultra-processed foods and greater environmental impact in Brazil (11, 12) as well as lower adherence to sustainable diets (13). 

These data were essential for monitoring the quality of the Brazilian diet and informing policies on food and nutrition. They supported the composition of the current basic market basket (14) and strengthened policy guidelines already in force such as the National School Meals Program (*Programa Nacional de Alimentação Escolar*) (15), all of which are based on the Dietary Guidelines. 

Furthermore, protocols were drawn up for using the *Dietary Guidelines for the Brazilian Population* in dietary guidance (16). These documents focus on each stage or event in the life cycle and serve as the foundation for the dietary guidance provided by Primary Health Care professionals, optimizing the care and monitoring of individuals utilizing the Brazilian National Health System (*Sistema Único de Saúde*). The content of the protocols was largely based on the dietary profile of the Brazilian population described by the Household Budget Survey, supporting the dissemination of recommendations for healthy eating and making the Guidelines’s messages more accessible to the population.

We acknowledge that the methodology presented may lead to a slight overestimation of the share of ready-to-eat foods in the population’s diet. This occurs due to the decision to maintain as individual non-disaggregated items those foods whose main components, or those with the greatest calorie contribution, belong to the same group in the Nova classification. As a result, the majority of items with some ambiguity were classified as ultra-processed foods. Sensitivity analysis identified significant differences only between the processed and ultra-processed food groups, with small magnitude.

As a strength, this study presented a systematic method for classifying food consumption databases according to the Nova classification, bringing transparency to the results. The methodology was effective in classifying the wide diversity of foods and culinary preparations consumed in Brazil, covering dietary practices in all macro-regions of the country, as well as urban and rural areas, according to the Survey sample. The efficiency and scope of this methodology suggests its potential for replication in other food consumption studies carried out in Brazil. Among the study’s limitations, the possibility of a slight overestimation of the consumption of ultra-processed foods at the expense of processed foods stands out, which can be mitigated through sensitivity analyses. In studies involving health outcomes, sensitivity analyses are essential to ensure that classification uncertainties do not cause significant changes in the associations found.

Despite the effectiveness of applying this classification method, use of instruments specifically developed to understand food consumption according to the degree of food processing can improve estimates for future surveys. Examples of instruments are the Nova 24-hour food recall instrument (17), the Nova Score instrument (18) and the Nova food frequency questionnaire (19), available on the QuestNova platform (https://questnova.com.br).

Food consumption analyses based on the Nova classification are effective ways of monitoring the population’s adherence to the recommendations of the *Dietary Guidelines for the Brazilian Population*. Using a standardized method for categorizing the Household Budget Survey according to the Nova classification was effective and led to estimates whose uncertainties minimally affected the results. This action strengthens food and nutritional surveillance as applied to the Dietary Guidelines.

## Data Availability

The databases and the analysis codes used in this research are available at: https://www.ibge.gov.br/estatisticas/sociais/saude/24786-pesquisa-de-orcamentos-familiares-2.html?edicao=28523&t=downloads.
