# Supplementary material for: Application of the Nova food classification to the 2017-2018 Household Budget Survey: monitoring adherence to the recommendations of the Dietary Guidelines for the Brazilian Population
Source: Epidemiol Serv Saude. 2025 May 12;34:e20240369. doi: 10.1590/S2237-96222025v34e20240369.en (PMC12077547; doi:10.1590/S2237-96222025v34e20240369.en)
Supplement: Translation

# Translation: Aplicação da classificação de alimentos Nova na Pesquisa de Orçamentos Familiares 2017-2018: estratégia de monitoramento da adesão às recomendações do Guia Alimentar para a População Brasileira

**DOI:** 10.1590/S2237-96222025v34e20240369.pt

**Authors:** Gabriela Lopes da Cruz, Giovanna Calixto Andrade, Fernanda Rauber, Renata Bertazzi Levy, Maria Laura da Costa Louzada

**Affiliations:** 1 Universidade de São Paulo, Faculdade de Saúde Pública, Departamento de Nutrição, São Paulo, SP, Brasil; 2 Universidade de São Paulo, Núcleo de Pesquisas Epidemiológicas em Nutrição e Saúde, São Paulo, SP, Brasil; 3 Universidade de São Paulo, Faculdade de Medicina, Departamento de Medicina Preventiva, SP, Brasil

**Keywords:** Vigilância Alimentar e Nutricional, Inquéritos Nutricionais, Guias Alimentares, Classificação Nova, Estudos Transversais

## Abstract

**Objetivo:**

Descrever o método de categorização dos alimentos da Pesquisa de Orçamentos Familiares 2017-2018 conforme a classificação Nova, trazendo transparência e replicabilidade ao processo de monitoramento da adesão às recomendações do *Guia Alimentar para a População Brasileira*.

**Métodos:**

Tratou-se de classificação dos alimentos reportados na pesquisa em quatro fases. Foram elas: identificação das preparações culinárias e itens compostos por mais de um alimento, determinação do receituário das preparações culinárias e dos itens a serem desagregados, aplicação da classificação Nova e análise de sensibilidade.

**Resultados:**

Após a desagregação, 1.856 itens foram classificados segundo a classificação Nova, sendo 1.050 alimentos in natura ou minimamente processados, 54 ingredientes culinários processados, 160 alimentos processados e 592 alimentos ultraprocessados. Alimentos cuja classificação gerou dúvidas representaram 4% da energia da dieta. A participação calórica de ultraprocessados variou de 19,7% (intervalo de confiança de 95% [IC95%] 19,3; 20,1) para 17,7% (IC95% 17,4; 18,1) após análise de sensibilidade.

**Conclusão:**

O uso de método padronizado para aplicar a classificação Nova à Pesquisa de Orçamentos Familiares foi efetivo e levou a estimativas cujas dúvidas impactaram minimamente o resultado. A metodologia apresentada pode ser replicada em futuras edições da pesquisa e demais estudos de consumo alimentar, fortalecendo a vigilância alimentar e nutricional aplicada ao guia alimentar.

## Introdução

A Pesquisa de Orçamentos Familiares 2017-2018 (1) apresentou, pela primeira vez, o relatório do consumo alimentar pessoal da população brasileira, com base na classificação de alimentos Nova (2). Essa classificação divide os alimentos em quatro grupos de acordo com a extensão e o propósito do processamento ao qual foram submetidos. Os grupos são: alimentos in natura ou minimamente processados, ingredientes culinários processados, alimentos processados e alimentos ultraprocessados.

A avaliação do consumo alimentar segundo a classificação Nova permite o monitoramento da adesão da população às recomendações do *Guia Alimentar para a População Brasileira* (3), publicado em 2014. Este preconiza “Prefira sempre alimentos in natura ou minimamente processados e preparações culinárias a alimentos ultraprocessados” (3). A adesão às recomendações do guia beneficia a saúde da população, contribui para a prevenção de doenças crônicas e promove a sustentabilidade ambiental. O guia é o instrumento oficial do Ministério da Saúde que embasa políticas públicas de alimentação e nutrição e orienta ações nos serviços de saúde do país, o que torna a avaliação de sua adesão fundamental.

Inquéritos alimentares que realizam coleta de dados utilizando recordatórios de 24 horas inespecíficos para captar informações sobre o grau de processamento industrial dos alimentos apresentam desafios para a classificação de alguns itens segundo a classificação Nova. Nesses casos, é necessário desagregar as preparações culinárias (refeições elaboradas a partir de alimentos minimamente processados e ingredientes culinários, feitas em casa ou em restaurantes) em seus ingredientes subjacentes, para que estes ingredientes individuais, ao invés da preparação inteira, sejam classificados. Alguns itens alimentares, a depender de sua composição, podem ser atribuídos a diferentes grupos da classificação Nova (como pães e queijos, que podem ser processados ou ultraprocessados), o que também constitui um desafio.

Considerando a crescente relevância da classificação Nova e a amplitude de possibilidades de uso dos dados da Pesquisa de Orçamentos Familiares, é esperado que diversos estudos sejam realizados utilizando-as como ferramentas de análise. Prevê-se a aplicação da classificação Nova em outras edições da pesquisa, assim como em demais inquéritos alimentares. O objetivo deste artigo foi descrever o método de categorização dos alimentos da Pesquisa de Orçamentos Familiares 2017-2018 conforme a classificação Nova. Buscou-se trazer transparência e replicabilidade ao processo de monitoramento da adesão às recomendações do Guia Alimentar para a População Brasileira.

## Métodos

### Delineamento

Trata-se da descrição e avaliação da metodologia utilizada para classificar a Pesquisa de Orçamentos Familiares 2017-2018, de acordo com a classificação Nova. 

### Contexto e tamanho do estudo

Utilizaram-se dados do módulo de consumo alimentar pessoal da Pesquisa de Orçamentos Familiares, realizada pelo Instituto Brasileiro de Geografia e Estatística entre julho de 2017 e julho de 2018. Na pesquisa, foi realizado processo de amostragem complexa em dois estágios. No primeiro estágio, houve o agrupamento de setores censitários com estratificação geográfica e socioeconômica seguida de sorteio e, no segundo estágio, sorteio de domicílios pertencentes aos setores selecionados. Detalhes sobre a pesquisa podem ser encontrados em publicação anterior (1).

Os bancos de dados das Pesquisas de Orçamentos Familiares estão disponíveis em: https://www.ibge.gov.br/estatisticas/sociais/saude/24786-pesquisa-de-orcamentos-familiares2.html?edicao=28523&t=downloads. 

### Participantes e variáveis

Informações sobre o consumo alimentar individual foram coletadas em subamostra de 20.112 domicílios, com informações fornecidas por moradores de 10 anos ou mais de idade. No total, 46.164 indivíduos foram selecionados para responder a inquéritos recordatórios de 24 horas em dois dias não consecutivos. Os participantes foram questionados sobre todos os alimentos e as bebidas consumidos no dia anterior à entrevista. Após identificar os itens consumidos, os entrevistadores solicitaram detalhes sobre o tipo de preparação de cada alimento (quando aplicável) e a quantidade consumida. Foram registrados 1.591 itens alimentares. Considerando as diferentes formas de preparo associadas a esses itens (1 – assado, 2 – cozido com gordura, 3 – cozido sem gordura, 4 – cru, 5 – empanado/à milanesa, 6 – ensopado, 7 – frito, 8 – grelhado/brasa/churrasco, 9 – refogado e 99 – não determinado), o número de itens alimentares reportados chegou a 2.534. 

### Mensuração

A categorização dos alimentos segundo a classificação Nova foi realizada em quatro fases, aplicadas por dois nutricionistas pesquisadores da área de consumo alimentar familiarizados com as Pesquisas de Orçamentos Familiares. Em todas as fases, as dúvidas acerca da classificação foram discutidas conjuntamente com demais pesquisadores conhecedores da classificação Nova e dos hábitos alimentares brasileiros até se alcançar consenso. 

### Fase 1 – Identificação das preparações culinárias e itens compostos por mais de um alimento

A primeira fase consistiu na identificação de itens que deveriam ser desagregados para serem classificados, de forma a discriminar os ingredientes que os compõem (4). A desagregação é necessária para classificar os ingredientes individuais de preparações culinárias (como feijoada, baião de dois e bolos, e preparações do tipo refogado, frito e empanado) e conjuntos de alimentos (itens compostos, como cuscuz com leite e açaí com guaraná e granola) de acordo com a classificação Nova. 

Foram identificadas para desagregação preparações culinárias típicas da alimentação brasileira (como arroz, feijão, macarronada, moqueca etc.) e alimentos cuja forma de preparo envolvesse a adição de ingredientes (cozido com gordura, empanado/à milanesa, ensopado, frito e refogado). Não foram selecionados para desagregação alimentos com um único ingrediente nas formas cru, assado e cozido sem gordura. 

Foram exemplos de itens selecionados para desagregação:

Arroz (polido, parboilizado, agulha, agulhinha)
Tipo: preparação culinária
Desagregado nos itens: arroz, alho, cebola, óleo de soja e sal
Suco de maracujá
Tipo: preparação culinária
Desagregado nos itens: maracujá, água e açúcar
Café com leite
Tipo: item composto
Desagregado nos itens: café e leite

Itens que necessitam desagregação foram diferenciados de produtos industrialmente fabricados (um alimento composto por vários ingredientes pré-combinados e processados pela indústria), que podem ser alimentos processados ou ultraprocessados. Esses itens foram identificados com informações presentes na nomenclatura, como marca do alimento (Tang e Ovomaltine) e termos “diet” e “light”, “pronto para consumo” e “industrializado”. Mesmo compondo múltiplos ingredientes, tais itens não são desagregados, alocados portanto para a fase 3 para serem classificados segundo a classificação Nova (2). Como exemplo, farofa pronta e farofa pronta light foram identificadas como itens industrializados e selecionados para a fase 3. Farofa com ovos, farofa de banana e farofa de couve foram categorizadas como preparações culinárias e selecionadas para desagregação. 

Para itens com descrição inespecífica, foram realizadas consultas a fontes externas para determinar a alternativa mais provável. A principal conduta nesses casos foi comparar nessas bases a frequência da disponibilidade de alimentos que poderiam ser tanto preparações culinárias quanto produtos industrialmente fabricados, identificando aqueles de maior ocorrência. Exemplos de fontes externas incluíam:

consulta a dados de compras de alimentos do módulo de disponibilidade domiciliar de alimentos no Brasil (5) realizado no mesmo período, comparando disponibilidade e frequência de compras dos mesmos alimentos em diferentes formas de consumo. Exemplo: para lasanha, comparou-se a frequência de aquisição de “massa de lasanha” – indicativo do preparo caseiro de lasanha – versus aquisição de “lasanha congelada” – indicativo de alimento industrializado.
consulta a sites de venda de alimentos em redes de varejo, verificando a disponibilidade dos mesmos alimentos em diferentes graus de processamento. Exemplo: para sucos, averiguou-se a presença/ausência no varejo de itens como suco de tamarindo, suco de cupuaçu, suco de beterraba e suco de couve engarrafados ou em embalagens assépticas. A ausência/baixa frequência foi indicativo para categorizá-los como preparação culinária feita a partir da fruta.
verificação dos grupos da classificação Nova aos quais pertenceriam os ingredientes de um item alimentar elegível para desagregação caso fosse desagregado, dispensando a necessidade de desagregação se todos pertencessem ao mesmo grupo. Exemplo: o item hambúrguer, composto por pão de hambúrguer, hambúrguer bovino, ketchup e maionese – todos alimentos ultraprocessados – dispensou desagregação.

### Fase 2 – Determinação do receituário das preparações culinárias e dos itens a serem desagregados

Preparações culinárias e itens compostos selecionados na fase 1 foram desagregados nos ingredientes que os compõem e correspondentes quantidades, utilizando receitas padronizadas da Tabela Brasileira de Composição de Alimentos da Universidade de São Paulo, versão 7.0 (disponível em: http://www.fcf.usp.br/tbca). 

### Fase 3 – Aplicação da classificação Nova

Todos os alimentos (incluindo itens que não foram desagregados e ingredientes subjacentes àqueles desagregados) foram categorizados de acordo com a classificação Nova nos seus quatro grupos: alimentos in natura ou minimamente processados, ingredientes culinários processados, alimentos processados e alimentos ultraprocessados (2). Para isso, utilizaram-se definições e exemplos (Tabela 1) conforme Monteiro e colaboradores (2).

**Tabela 1:** Definição e exemplos dos grupos de alimentos da classificação Nova, de acordo com o grau e propósito do processamento industrial

Grupo da classificação Nova	Definição	Exemplos
**Alimentos in natura ou minimamente processados**	Alimentos in natura: partes comestíveis de plantas, animais, fungos e algas, que passaram por pouca ou nenhuma alteração em relação ao estado que se encontram na natureza. Minimamente processados: alimentos in natura que passaram por modificações mínimas, que incluem remoção de partes não comestíveis, secagem, trituração, moagem, torrefação, cozimento em água, pasteurização, refrigeração, congelamento, embalagem ou embalagem a vácuo. Vitaminas e minerais podem ser adicionados a alimentos deste grupo, geralmente para substituir nutrientes perdidos durante o processamento.	Cereais, leguminosas, frutas, sementes, folhas, caules, raízes e tubérculos, carnes frescas (músculos e vísceras), ovos, leite, farinhas. Também incluem alimentos compostos por dois ou mais itens deste grupo, como frutas secas mistas, granola feita de cereais, nozes e frutas secas sem adição de açúcar, mel ou óleo, macarrão e massas sem recheio, cuscuz e polenta feitos com farinhas, flocos ou farelos e água, e alimentos fortificados, como farinhas fortificadas com ferro e ácido fólico.
**Ingredientes culinários processados**	Substâncias obtidas diretamente de alimentos do primeiro grupo ou da natureza por meio de processos como prensagem, centrifugação, extração, refino, desidratação e mineração. Esses processos produzem ingredientes utilizados para temperar e preparar alimentos do primeiro grupo, criando preparações culinárias. Aditivos geralmente não são necessários e raramente são encontrados neste grupo.	Açúcar (cristal, refinado, mascavo e demerara), melado de cana, mel, óleos vegetais, gorduras animais (como manteiga e banha), amido de milho, vinagre e sal. Também incluem produtos compostos por dois itens deste mesmo grupo, como manteiga com sal, e itens adicionados de vitaminas ou minerais, como sal iodado.
**Alimentos processados**	Incluem produtos produzidos pela adição de sal, açúcar, óleos ou gorduras a alimentos in natura ou minimamente processados, constituíndo, portanto, a combinação de alimentos dos dois primeiros grupos. São produtos de processamento relativamente simples, como conservação em lata e vidro, além de fermentação não alcoólica no caso de queijos e pães, este último seguido de assamento. Neste grupo, o processamento visa aumentar a durabilidade e complexificar as qualidades sensoriais dos alimentos. Alimentos processados frequentemente contêm aditivos que prolongam a duração do produto, preservam suas propriedades ou inibem a proliferação de microrganismos (como conservantes e antioxidantes). Não contêm aditivos com função cosmética.	Pães, queijos, leguminosas e cereais em conserva de salmoura, nozes e sementes salgadas ou açucaradas, frutas em calda e geleias. Carnes secas e curadas, peixes secos ou enlatados. Bebidas alcoólicas fermentadas como cerveja e vinho. Quando feitos exclusivamente com alimentos dos dois primeiros grupos, pastéis, bolos, biscoitos, produtos prontos para aquecer, como hambúrgueres e tortas, massas e pizzas pré-preparadas.
**Alimentos ultraprocessados**	Formulações industriais altamente processadas, resultantes de diversas etapas e tipos de processamento. São marcadores de alimentos ultraprocessados: a) aditivos alimentares cosméticos que modificam as características sensoriais do produto, como aromatizantes, corantes, adoçantes artificiais, emulsificantes, espessantes, realçadores de sabor e emulsionantes; e/ou b) substâncias de uso industrial, raramente utilizadas na culinária, como frutose, xarope de milho com alto teor de frutose, açúcar invertido, maltodextrina, dextrose, lactose, glúten adicionado, óleos hidrogenados ou interesterificados, proteínas hidrolisadas, isolado de proteínas e carne mecanicamente separada.	Refrigerantes, refrescos de fruta, energéticos, bebidas lácteas e iogurtes aromatizados. Pães de forma, biscoitos doces e salgados. Balas, chocolates e doces em geral, margarina, nuggets, salsichas, hambúrgueres, embutidos e outros produtos de carne reconstituída, cereais de café da manhã extrusados, macarrão instantâneo, sopas em pó, tempero pronto. Produtos prontos para aquecer, como hambúrgueres, tortas, massas e pizzas pré-preparadas.

Itens alimentares correspondentes a um único grupo da classificação Nova, como frutas, verduras e legumes in natura, ovos, carnes frescas, óleo vegetal, refrigerantes, margarina, bolachas recheadas e sucos artificiais, foram atribuídos ao grupo equivalente. Para outros alimentos que poderiam apresentar variações, informações contidas no campo de descrição do alimento foram utilizadas para tomada de decisão. 

Como exemplo, alimentos cuja nomenclatura indicava fresco ou in natura foram alocados para o grupo in natura ou minimamente processados (atum fresco, cogumelo in natura, amendoim em grão in natura). Alimentos em conserva foram classificados como processados (milho verde, palmito e sardinha em conserva). Itens com terminologias “diet” e “light”, “pronto para consumo” e “industrializado” foram classificados como ultraprocessados. 

Iogurte natural e iogurte desnatado foram alocados no grupo in natura ou minimamente processados. Iogurte de qualquer sabor, iogurte de qualquer sabor light e iogurte de qualquer sabor diet foram classificados como ultraprocessados. Pão de sal e pão caseiro foram identificados como alimentos processados. Pão de forma industrializado de qualquer marca, pão diet (de forma industrializado) e pão light (de forma industrializado) foram classificados como ultraprocessados. Quando a nomenclatura do item especificava a marca do alimento, buscou-se a lista de ingredientes para classificá-lo. 

Outra estratégia foi utilizar informações de sites de vendas de alimentos para orientar a classificação de itens que, a depender de sua composição, poderiam pertencer a diferentes grupos da classificação Nova (como queijos, leite de coco e bacon, que podem ser processados ou ultraprocessados). Fez-se levantamento das listas de ingredientes de diferentes marcas do mesmo alimento disponíveis no varejo, atribuindo a classificação ao grupo da Nova mais frequente entre elas. Por meio dessa abordagem, itens como creme de leite e granola foram classificados como ultraprocessados devido à presença de substâncias de uso industrial e aditivos cosméticos (Tabela 1) na maioria dos produtos à venda no mercado brasileiro. 

Características da cultura e hábitos alimentares brasileiros também foram consideradas na tomada de decisão, optando por classificar o alimento de acordo com a alterativa mais habitual. Foi o caso dos doces de frutas, como marmelada, goiabada e doce de banana em pasta, todos classificados como alimentos processados.

### Fase 4 – Análise de sensibilidade

Após consulta a fontes externas e discussão em grupo, alimentos ainda considerados de classificação incerta nas fases 1 e 3 foram submetidos à decisão em grupo para a classificação. Esses alimentos foram selecionados para análises de sensibilidade, avaliando como a variação da classificação poderia afetar os resultados. Realizou-se a avaliação das diferenças nas estimativas com base na sobreposição dos intervalos de confiança.

## Análise estatística

Utilizaram-se informações do primeiro dia de recordatório alimentar da pesquisa, uma vez que este apresentou melhor qualidade na coleta de dados e foi o padrão de análise adotado no relatório do Instituto Brasileiro de Geografia e Estatística (1). As quantidades de cada alimento e bebida reportadas foram convertidas em gramas ou mililitros e em calorias utilizando a Tabela Brasileira de Composição de Alimentos. A participação calórica dos alimentos no total energético da dieta foi calculada. A análise de sensibilidade reclassificou os alimentos cuja classificação apresentou dúvidas, alocando-os no grupo alternativo à decisão final, e avaliou a sobreposição dos intervalos de confiança das médias. 

## Resultados

As quatro fases delineadas na metodologia, juntamente com seus resultados, foram ilustradas no fluxograma (Figura 1).

**Figura 1:** Método de categorização dos alimentos da Pesquisa de Orçamentos Familiares de acordo com a classificação Nova. Brasil, 2017-2018 (n=46.164)

Na fase 1, foram selecionados 1.241 itens (49%) para desagregação, enquanto 1.293 itens (51%) permaneceram não desagregados e foram diretamente alocados para a fase 3. Entre aqueles selecionados para desagregação, 77 foram considerados dúvidas devido à impossibilidade de identificá-los claramente como preparações culinárias ou produtos industrialmente fabricados. Esses itens, somados, representaram 2,2% (IC95% 1,9; 2,6) do total de calorias consumidas pela população brasileira e incluíram: doces variados (n=6), pastéis (n=5), pizzas (n=15), refeições prontas de massa (lasanha, caneloni) (n=15), tortas e salgados (coxinha, empada, croquete, quibe) (n=15), sanduíches (n=20) e molhos (n=1) (Tabela 2). 

**Tabela 2:** Itens alimentares reportados na Pesquisa de Orçamentos Familiares identificados como dúvidas nas fases 1 e 3 da metodologia. Brasil, 2017-2018 (n=46.164)

**Itens identificados como “Dúvida”**	
**Fase 1** – **Identificação das preparações culinárias e itens compostos por mais de um alimento** (n=77)	**Fase 3** – **Aplicação da classificação Nova** (n=28)
Americano Bolinha de queijo Bolinho de aipim Bolinho de bacalhau Brigadeiro Canelone de frango Canelone de queijo Canelone de queijo e presunto Canelone de ricota Capeletti de carne Capeletti de frango Coxinha Croquete Doce de amendoim Doce de massa folhada Empada (queijo, carne, camarão etc.) Empadão (queijo, frango, camarão, palmito etc.) Esfirra Esfirra de carne Esfirra de frango Esfirra de queijo Esfirra de ricota Lasanha quatro queijos Maria-mole Merengue Mil folhas Minipastel Molho de tomate Pastel com recheio doce Pastel de forno integral/pastel assado integral (queijo, carne, palmito etc.) Pastel de forno/pastel assado (queijo, carne, palmito etc.) Pastel de nata Pizza Pizza de calabresa Pizza de frango com catupiry Pizza de lombinho defumado Pizza de muçarela de búfala e tomate seco Pizza de peixe (atum, salmão, sardinha) Pizza doce de qualquer sabor Pizza marguerita (muçarela, tomate e manjericão) Pizza de muçarela Pizza napolitana Pizza portuguesa Pizza de presunto Pizza vegetariana Pizza de pepperoni Pizza de queijos Quibe Rizole (queijo, carne, camarão etc.) Rondele/rondeli/rondelle Rondele de camarão Rondele de carne Rondele de queijo e presunto Sanduíche bauru Sanduíche de atum Sanduíche de carne assada Sanduíche de carne de porco Sanduíche de filé Sanduíche de frango Sanduíche de mortadela Sanduíche de peito de peru Sanduíche de presunto Sanduíche de queijo (não especificado) Sanduíche de queijo minas Sanduíche de queijo prato Sanduíche de queijo prato com presunto Sanduíche de rosbife Sanduíche de salame Sanduíche de sardinha Sanduiche natural Sanduíche de presunto, queijo e tomate Sanduíche vegetariano Tortas salgadas de qualquer sabor Yakissoba de camarão Yakissoba de carne Yakissoba de frango Yakissoba de legumes	**Itens individuais não desagregados:** Amendoim amanteigado Creme de arroz Creme de leite Geleia de frutas de qualquer marca ou sabor Granola Leite de coco Marshmallow Mix de cereais Molho de tomate Paçoquinha de amendoim Pão de milho Pão de queijo Quebra-queixo Suco Suco de goiaba Suco de laranja Suco de manga Suco de pêssego Suco de pêssego em calda Suco de uva Torrão de amendoim **Após desagregação**: Caju, suco, concentrado, envasado Creme de leite Laranja, pera, suco, sem açúcar Laranja, suco, sem açúcar, Citrus sinensis Leite, condensado, com açúcar, enlatado Molho, pimenta, tabasco Uva, suco, concentrado, envasado

Após discussão em grupo, os itens foram mantidos majoritariamente como não desagregados, com base na premissa de que seus principais componentes, ou aqueles com maior contribuição calórica, pertenceriam ao mesmo grupo da classificação Nova, justificando sua análise conjunta.

Na fase 2, preparações culinárias e itens compostos foram desagregados utilizando receitas padronizadas, resultando em 7.356 ingredientes, sendo 563 ingredientes únicos sem repetição. Isso foi esperado, uma vez que os mesmos ingredientes estavam presentes em diferentes receitas.

Na fase 3, após a desagregação, 1.856 itens foram organizados de acordo com a classificação Nova em seus quatro grupos, sendo 1.050 (56,6%) identificados como alimentos in natura ou minimamente processados, 54 (2,9%) como ingredientes culinários processados, 160 (8,6%) como alimentos processados e 592 (31,9%) como alimentos ultraprocessados. 

Para 28 itens, que representaram 1,8% (IC95% 1,7; 1,9) do total calórico consumido na pesquisa, informações na nomenclatura dos alimentos foram insuficientes para identificar características do processamento industrial, sendo alocados para dúvidas. Esses itens compreenderam: sucos de frutas (n=11), cereais matinais (granola e mix de cereais) (n=3), doces variados (n=7), alguns tipos de pães (n=2) e molhos (n=5) (Tabela 2). 

A natureza dessas dúvidas derivou da falta de especificações na descrição dos itens da pesquisa quanto ao grau de processamento. Isso foi especialmente relevante para identificar corretamente itens como creme de leite fresco ou industrializado e sucos naturais ou industrializados. Outra dúvida decorreu do fato de que alguns alimentos, como geleias e molhos prontos, podem pertencer a diferentes grupos da classificação Nova a depender da lista de ingredientes, que varia entre marcas e pode determinar a classificação como processado ou ultraprocessado.

Também geraram dúvidas os alimentos cuja descrição incluía termos como “não especificado”, quando da incerteza da condição do alimento (se fresco, congelado ou enlatado), e, quando desconhecido, se adoçado/salgado. Itens que geraram dúvidas foram majoritariamente classificados como ultraprocessados. Alimentos descritos como “não especificado” foram classificados tal qual o item semelhante de consumo mais frequente. Como exemplo, pão “não especificado” foi classificado da mesma forma que pão francês, o pão mais consumido na pesquisa, no grupo de alimentos processados. 

Na fase 4, os itens identificados como dúvidas nas fases 1 e 3 foram selecionados para análise de sensibilidade. Utilizando a metodologia descrita, segundo a classificação Nova a participação de alimentos in natura ou minimamente processados foi 53,4% (IC95% 53,0; 53,8), ingredientes culinários processados foi 15,6% (IC95% 15,4; 15,8), alimentos processados foi 11,3% (IC95% 11,1; 11,5) e alimentos ultraprocessados foi 19,7% (IC95% 19,3; 20,1) do total de calorias ingeridas pela população brasileira. 

A análise de sensibilidade consistiu na reclassificação de todos os alimentos que geraram dúvidas para o grupo da classificação não selecionado. Após essa análise, a participação de alimentos in natura ou minimamente processados foi 54,1% (IC95% 53,7; 54,4), ingredientes culinários processados foi 15,8% (IC95% 15,6; 16,0), alimentos processados foi 12,4% (IC95% 12,2; 12,7) e alimentos ultraprocessados foi 17,7% (IC95% 17,4; 18,1) da ingestão calórica da população (Figura 2).

**Figura 2:** Resultados da análise de sensibilidade considerando a participação de grupos de alimentos de acordo com a classificação Nova no total de calorias consumidas pela população brasileira com 10 anos ou mais de idade. Brasil, 2017-2018 (n=46.164)

As principais mudanças na análise de sensibilidade incluíram a troca de alimentos do grupo ultraprocessado para processado. Houve aumento na quantidade de sucos de fruta naturais e diminuição na quantidade de sucos artificiais. Todos os sanduíches foram desagregados, reduzindo a categoria de lanches ultraprocessados. Observou-se queda na quantidade de doces ultraprocessados, que na análise de sensibilidade foram selecionados para desagregação.

## Discussão

A utilização de um método padronizado foi eficaz para categorizar a Pesquisa de Orçamentos Familiares segundo a classificação Nova, mesmo a pesquisa realizando coleta de dados com recordatório inespecífico para captar o grau de processamento dos alimentos. Essa metodologia, aplicada por uma equipe conhecedora dos hábitos alimentares brasileiros, resultou em poucas dúvidas, impactando minimamente os resultados obtidos.

A vigilância alimentar e nutricional é parte fundamental da Política Nacional de Alimentação e Nutrição. Visa-se gerar dados para acompanhamento e avaliação contínua do perfil alimentar e nutricional da população, essenciais para informar políticas públicas e orientar ações nos serviços de saúde. A Pesquisa de Orçamentos Familiares se destaca como importante base de dados por sua representatividade nacional, assegurada pela participação de mais de 40 mil indivíduos de áreas urbanas e rurais de diversas regiões do país. 

Com a publicação do *Guia Alimentar para a População Brasileira* em 2014, esse inquérito se tornou instrumento fundamental para monitorar a adesão da população às suas recomendações por meios de análises utilizando a classificação Nova. Espera-se que a metodologia apresentada neste estudo seja replicada nas futuras edições da pesquisa, dando continuidade ao processo de monitoramento. 

As Pesquisas de Orçamentos Familiares contam com diferentes conjuntos de dados de alimentação, como consumo alimentar individual, disponibilidade domiciliar de alimentos e segurança alimentar, e informações socioeconômicas bem caracterizadas. A combinação desses dados possibilita abordagens sistêmicas de estudo de alimentação, necessidade cada vez mais urgente para enfrentar difíceis problemas de saúde global, como obesidade, desnutrição e mudanças climáticas (6). 

Análises e discussões da alimentação baseadas na classificação Nova têm ganhado crescente relevância. Evidências consistentes apontaram associação entre o consumo de alimentos ultraprocessados e o risco de mais de 30 desfechos em saúde, incluindo obesidade e demais doenças crônicas não transmissíveis, além de mortalidade por todas as causas (7). Esse problema é particularmente importante no Brasil devido à tendência global de aumento do consumo de ultraprocessados em países populosos e de média-renda, em particular na América Latina (8).

O método empregado neste estudo permitiu avaliar detalhadamente o consumo de alimentos ultraprocessados em diferentes grupos populacionais. Essa abordagem pode ser replicada para entender melhor padrões de consumo e suas implicações na saúde pública. A padronização desse método em diversos contextos pode ajudar a identificar variáveis socioeconômicas que influenciam o consumo alimentar, contribuindo para a elaboração de estratégias de promoção da saúde mais eficazes.

A metodologia descrita para identificação de alimentos segundo a classificação Nova nas Pesquisas de Orçamentos Familiares foi aplicada para geração de dados de monitoramento da qualidade da alimentação da população brasileira. Dados de aquisição domiciliar de alimentos revelaram tendência crescente da disponibilidade de alimentos ultraprocessados no Brasil nas últimas três décadas, concomitante à queda de alimentos in natura e minimamente processados (9). Dados de consumo alimentar evidenciaram que alimentos ultraprocessados correspondem a um quinto da dieta brasileira, com diferenças significativas no consumo segundo fatores socioeconômicos (10). Também utilizando essa metodologia, foi identificada associação entre o consumo de ultraprocessados e maior impacto ambiental no Brasil (11,12) e a menor adesão a dietas sustentáveis (13). 

Esses dados foram fundamentais para monitorar a qualidade da dieta brasileira e informar políticas públicas de alimentação e nutrição. Eles apoiaram a composição da atual cesta básica de alimentos (14) e fortaleceram diretrizes de políticas já em vigor como o Programa Nacional de Alimentação Escolar (15), todas fundamentadas pelo guia alimentar. 

Ademais, foram elaborados Protocolos de uso do *Guia Alimentar para a População Brasileira* na orientação alimentar (16). Esses documentos focaram cada fase ou evento do ciclo de vida para qualificar as orientações alimentares realizadas por profissionais das equipes de Atenção Primária à Saúde, otimizando o atendimento e acompanhamento dos usuários do Sistema Único de Saúde. O conteúdo dos protocolos foi amplamente baseado no perfil alimentar brasileiro descrito pela Pesquisa de Orçamentos Familiares, apoiando a disseminação das recomendações para uma alimentação saudável e tornando as mensagens do guia mais acessíveis à população.

Reconhece-se que a metodologia apresentada pode levar à leve superestimação da participação de alimentos prontos para consumo na dieta. Isso ocorre devido à decisão de manter como itens individuais não desagregados os alimentos cujos principais componentes, ou aqueles com maior contribuição calórica, pertencem ao mesmo grupo da classificação Nova. Como resultado, a maioria dos itens que apresentavam alguma ambiguidade foi classificada como alimento ultraprocessado. Análise de sensibilidade identificou diferenças significativas apenas entre os grupos de alimentos processados e ultraprocessados, com pequena magnitude.

Como fortaleza, este estudo apresentou um método sistemático para classificação de bancos de dados de consumo alimentar de acordo com a classificação Nova, trazendo transparência para os resultados. A metodologia foi eficaz para classificar a ampla diversidade de alimentos e preparações culinárias consumidos no Brasil, contemplando práticas alimentares de todas as macrorregiões do país, bem como áreas urbanas e rurais, conforme amostragem da pesquisa. A eficiência e abrangência dessa metodologia sugere o potencial para sua replicação em demais estudos de consumo alimentar realizados no Brasil. Entre as limitações, destaca-se a possibilidade de ligeira superestimação do consumo de ultraprocessados em detrimento dos alimentos processados, que pode ser atenuada com análises de sensibilidade. Em estudos com desfechos em saúde, análises de sensibilidade são fundamentais para assegurar que incertezas na classificação não provoquem alterações significativas nas associações observadas.

Apesar da efetividade de aplicar esse método de classificação, a utilização de instrumentos desenvolvidos especificamente para compreender o consumo alimentar de acordo com o grau de processamento pode melhorar estimativas de inquéritos alimentares futuros. São exemplos de instrumentos o recordatório alimentar 24h-Nova (17), o Escore Nova (18) e o questionário de frequência alimentar-Nova (19), disponibilizados na plataforma QuestNova (https://questnova.com.br).

Análises de consumo alimentar baseadas na classificação Nova são formas efetivas de monitorar a adesão às recomendações do *Guia Alimentar para a População Brasileira*. O uso de um método padronizado para a categorização da Pesquisa de Orçamentos Familiares de acordo com a classificação Nova foi eficiente e levou a estimativas cujas incertezas impactam minimamente os resultados. Essa ação fortalece a vigilância alimentar e nutricional aplicada ao guia alimentar.

## Data Availability

Os bancos de dados e os códigos de análise utilizados na pesquisa estão disponíveis em: https://www.ibge.gov.br/estatisticas/sociais/saude/24786-pesquisa-de-orcamentos-familiares-2.html?edicao=28523&t=downloads.
